# Association Between Clinic-Reported Third Next Available Appointment and Patient-Reported Access to Primary Care

**DOI:** 10.1001/jamanetworkopen.2022.46397

**Published:** 2022-12-13

**Authors:** Nishwa Shah, Lidija Latifovic, Christopher Meaney, Rahim Moineddin, Mary Beth Derocher, Mohammad Alhaj, Tara Kiran

**Affiliations:** 1 Department of Family and Community Medicine, Temerty Faculty of Medicine, University of Toronto, Toronto, Ontario, Canada; 2 MAP Centre for Urban Health Solutions in the Li Ka Shing Knowledge Institute, St Michael’s Hospital, Toronto, Ontario, Canada; 3 Dalla Lana School of Public Health, University of Toronto, Toronto, Ontario, Canada; 4 Institute for Clinical Evaluative Sciences, Toronto, Ontario, Canada; 5 Department of Family and Community Medicine, St. Michael’s Hospital, Toronto, Ontario, Canada; 6 Institute of Health Policy, Management and Evaluation, Toronto, Ontario, Canada

## Abstract

This cross-sectional study examines the association of the third next available appointment with patient-reported measures of access in primary care settings.

## Introduction

Timely access is one of the most interminable problems in primary care.^1^ Quality improvement efforts encourage practices to use third next available appointment (TNA) as a measure of access to physician appointments,^2^ but there has been no research to correlate this measure with patient-reported access. We used routinely collected data in a large primary care organization to examine the association between TNA and patient-reported access measures.

## Methods

Our study was conducted at the St Michael’s Hospital Academic Family Health Team, which has 6 clinics in Toronto, Canada, and has made efforts to improve access since 2010.^3^ Clerical staff collect TNA for staff physicians approximately biweekly. In 2014, the practice began distributing a monthly electronic patient experience survey.^4^ This initiative was deemed to require neither research ethics board approval nor written informed consent by institutional authorities at St Michael’s Hospital. Reporting followed the STROBE reporting guideline for observational studies.

We conducted a cross-sectional study to evaluate the association, at the physician level, between TNA and 5 patient-reported access measures collected during 2018 (eTable and eFigure in Supplement 1). We used random intercept linear regression models to account for clustering of physicians within sites and adjusted for physician gender, years in practice, and full-time equivalency. We ran models with TNA weighted for the inverse of the sample SD to account for variability throughout the year.

## Results

We analyzed data for 56 staff physicians (35 [62%] women) who had 1502 patient responses to the survey in 2018. The mean roster size was 644 (range, 139-2717), with mean (SD) 18.1 (10.8) years in practice, 0.6 (1.6) full-time equivalency, and TNA of 11.0 (6.0) days.

Most patients were women (920 [61%]), aged 35 to 79 years (1254 [83%]), rated their health as fair or poor (857 [57%]), were born in Canada (1011 [67%]), and had a college, university, or graduate degree (1306 [87%]). A total of 445 (30%) visited the clinic 5 or more times in the past year and 390 (26%) lived in the highest neighborhood income quintile.

There were few significant differences in access measures by clinic and physician characteristics (Table 1). Linear regression analysis showed that TNA was negatively correlated with patient-reported access measures; however, significance was noted only for timely access to the last booked appointment in both the unadjusted and adjusted models (Table 2).

**Table 1.** TNA and Patient-Reported Access Measures by Clinic and Physician Characteristics^a^

Physician characteristics	Mean (SD)					
	TNA, d	Timely access to the last booked appointment, %	Continuity with preferred physician, %	Ease of after-hours care, %	Same-day response to a telephone call, %	Same-/next-day access when sick, %
Clinic						
Site 1	12.0 (2.8)	82.67 (18.23)	82.36 (3.27)	37.65 (14.92)	60.93 (8.08)	70.28 (13.86)
Site 2	10.2 (66)	81.09 (11.91)	87.85 (11.17)	28.52 (15.67)	63.84 (14.24)	74.82 (14.45)
Site 3	11.4 (6.7)	74.96 (9.77)	87.49 (7.22)	38.30 (9.31)	54.44 (12.35)	69.51 (21.06)
Site 4	4.9 (2.5)	86.07 (7.25)	83.53 (9.79)	31.22 (8.47)	65.92 (13.80)	77.22 (16.04)
Site 5	12.9 (4.2)	71.42 (8.83)	83.18 (8.82)	28.32 (11.70)	57.19 (8.78)	74.74 (18.61)
Site 6	12.9 (4.5)	84.03 (11.21)	84.00 (5.71)	26.24 (9.50)	58.00 (15.86)	64.33 (15.52)
*P* value	.002	.04	.55	.07	.32	.47
Sex						
Male	9.0 (5.1)	81 (10)	85 (10)	31 (11)	62 (13)	70 (19)
Female	11.6 (5.7)	80 (13)	85 (7)	31 (12)	58 (13)	73 (17)
*P* value	.09	.93	.86	.50	.42	.96
Years of practice						
0-10	9.5 (6.8)	81 (12)	88 (8)	31 (14)	61 (10)	70 (17)
11-20	9.4 (4.3)	81 (12)	82 (9)	31 (10)	60 (17)	78 (15)
21-30	11.4 (4.4)	79 (14)	84 (7)	33 (15)	56 (15)	73 (12)
≥31	14.0 (5.9)	78 (8)	86 (7)	31 (10)	61 (9)	63 (25)
*P* value	.11	.90	.26	.99	.59	.59
Roster size						
<500	10.5 (4.2)	82 (14)	85 (8)	34 (12)	60 (16)	71 (22)
501-750	11.2 (6.5)	79 (11)	85 (9)	29 (12)	58 (11)	69 (17)
≥751	9.3 (5.6)	80 (10)	86 (6)	32 (12)	62 (14)	79 (9)
*P* value	.66	.59	.94	.44	.76	.20
FTE						
2-4	10.2 (4.1)	83 (13)	82 (8)	32 (10)	59 (17)	68 (24)
5-6	10.6 (5.9)	80 (12)	87 (8)	31 (11)	62 (12)	71 (18)
≥7	11.0 (6.5)	78 (11)	85 (8)	31 (14)	58 (12)	75 (11)
*P* value	.99	.56	.36	.83	.53	.80
Roster: FTE						
0-100	11.3 (5.8)	80 (12)	86 (8)	31 (14)	61 (15)	70 (21)
≥101	10.1 (5.5)	80 (11)	85 (8)	31 (10)	59 (12)	73 (15)
*P* value	.45	.97	.80	.85	.29	.50

Abbreviations: FTE, full-time equivalency; TNA, third next available appointment.

^a^ Wilcoxon test used for comparison of 2 groups; Kruskal-Wallis test used for comparison of more than 2 groups.

**Table 2.** Analyses of TNA Before and After Adjustment for Confounders^a^

Patient-reported access measure	Unadjusted weighted TNA^b^		Adjusted weighted TNA^b^^,^^c^	
	Regression coefficient (95% CI)	*P* value	Regression coefficient (95% CI)	*P* value
Timely access to the last booked appointment	−0.935 (−1.502 to −0.368)	.002	−1.035 (−1.633 to −0.437)	.001
Continuity with preferred physician	−0.258 (−0.727 to 0.211)	.29	−0.415 (−0.875 to 0.045)	.08
Ease of after-hours care	0.046 (−0.580 to 0.673)	.89	0.113 (−0.586 to 0.812)	.75
Same-day response to a telephone call	−0.610 (−1.284 to 0.063)	.08	−0.585 (−1.342 to 0.171)	.14
Same- or next-day access when sick	−0.686 (−1.628 to 0.255)	.16	−0.519 (−1.557 to 0.519)	.33

Abbreviation: TNA, third next available appointment.

^a^ Random intercept linear regression models were used to account for clustering of physicians within sites.

^b^ TNA weighted for the inverse of the sample SD.

^c^ Adjusted for sex, years in practice, and full-time equivalency.

## Discussion

We found that TNA had a significant inverse association with patient satisfaction with wait times for a regular appointment. As a TNA increased by 1 week, the proportion of patients reporting that the wait time for a regular appointment was good or excellent decreased by 7.35%. We found no association between TNA and continuity with their preferred physician, ease of after-hours access, same-day response to a telephone call, and same- or next-day access when sick.

Our findings are limited by sample size. The English-language, internet-based survey excluded perspectives of some populations and external generalizability is limited by the setting—an urban, academic practice in Canada with many physicians working part-time clinically.

The results were largely aligned with our hypotheses. Our practice approaches urgent appointments, urgent telephone calls, and after-hours care as a team with nurses supporting triage and physicians cross-covering. In contrast, access to a physician appointment for a routine visit is largely influenced by the physician’s availability. Contrary to our hypotheses, we did not observe an association between TNA and patient-reported ease for seeing their preferred physician. One explanation is patients choose to wait longer to see their preferred physician, valuing relationship over timeliness.^5^ Our results reinforce that timely access to primary care is a complex concept that is best assessed using multiple measures.^6^

## Notes

Supplement 1. **eTable.** Patient-Reported Access Measures Included in Our Study **eFigure.** Physicians Included in the Study

Supplement 2.
Data Sharing Statement

## References

[1] How Canada Compares. Results From the Commonwealth Fund’s 2020 International Health Policy Survey of the General Population in 11 Countries. Canadian Institute for Health Information; 2021.

[2] Murray M, Berwick DM. Advanced access: reducing waiting and delays in primary care. JAMA. 2003;289(8):1035-1040. doi:10.1001/jama.289.8.1035 12597760

[3] DeRocher M, Davie S, Kiran T. Using positive deviance to improve timely access in primary care. BMJ Open Qual. 2021;10(4):e001228. doi:10.1136/bmjoq-2020-001228 34649853 PMC8522670

[4] Slater M, Kiran T. Measuring the patient experience in primary care: comparing email and waiting room survey delivery in a family health team. Can Fam Physician. 2016;62(12):e740-e748. 27965350 PMC5154665

[5] Prentice JC, Davies ML, Pizer SD. Which outpatient wait-time measures are related to patient satisfaction? Am J Med Qual. 2014;29(3):227-235. doi:10.1177/1062860613494750 23939488

[6] Hempel S, Hilton LG, Stockdale S, . Defining access management in health care delivery organizations. J Ambul Care Manage. 2021;44(3):218-226. doi:10.1097/JAC.0000000000000382 34016848

