# Supplementary material for: Association Between Clinic-Reported Third Next Available Appointment and Patient-Reported Access to Primary Care
Source: JAMA Netw Open. 2022 Dec 13;5(12):e2246397. doi: 10.1001/jamanetworkopen.2022.46397 (PMC9856348; doi:10.1001/jamanetworkopen.2022.46397)
Supplement: Supplement 1. — eTable. Patient-Reported Access Measures Included in Our Study eFigure. Physicians Included in the Study [file jamanetwopen-e2246397-s001.pdf]

Supplementary Online Content

Shah N, Latifovic L, Meaney C, et al. Association between clinic-reported third next available appointment and patient-reported access to primary care. *JAMA Netw Open*. 2022;5(12):e2246397. doi:10.1001/jamanetworkopen.2022.46397

eTable. Patient-Reported Access Measures Included in Our Study

eFigure. Physicians Included in the Study

This supplementary material has been provided by the authors to give readers additional information about their work.

© 2022 Shah N et al. *JAMA Network Open.*

eTable: Patient-reported access measures included in our study

Patient-reported access measure	Outcome definition used in our study	Original Survey question	Survey Response options
Timely access to the last booked appointment	% of the physician's patients that reported the length of time it took between scheduling their most recent appointment and the visit itself was very good or excellent	Thinking about your most recent visit, on a scale of poor to excellent, how would you rate the length of time it took between making your appointment and the visit you just had?	5 = Excellent<br>4 = Very good<br>3 = Good<br>2 = Fair<br>1 = Poor
Continuity with preferred provider	% of the physician's patients that were able to see the physician or nurse practitioner that they prefer to see	How often do you see or speak to the physician or nurse practitioner that you prefer?	4 = Always<br>3 = Often<br>2 = Sometimes<br>1 = Rarely/never<br>0 = Does not have a preferred MD/NP
Ease of after-hours care	% of the physician's patients that reported it was somewhat or very easy to get care at the Family Health Team in the evening, weekend of holiday	Last time when you needed medical care in the evening, weekend, or holiday, how easy or difficult was it to get care at our family health team (i.e. without going to an outside walk-in clinic or emergency department?).	4 = Very easy<br>3 = Somewhat easy<br>2 = Somewhat difficult<br>1 = Very difficult<br>0 = Never needed care in the evening, weekends or holidays
Same day response to a phone call	% of the physician's patients that reported they were always/often able to get an answer the same day when they called the Family Health Team during regular practice hours	When you call our family health team with a medical question or concern during regular practice hours, how often do you get an answer the same day?	4 = Always<br>3 = Often<br>2 = Sometimes<br>1 = Rarely/never<br>0 = Never tried to contact via telephone
Same/next day access when sick	% of the physician's patients that reported they were seen by a doctor, nurse, or nurse practitioner on the same or next day when they were last sick and required care	Last time that you were sick, how quickly could you get to see a doctor, nurse, or nurse practitioner at our family health team?	6 = On the same day<br>5 = The next day<br>4 = In 2 to 3 days<br>3 = In 4 to 5 days<br>2 = In 6 to 7 days<br>1 = After more than 1 week<br>0 = Never able to get an appointment consultation

© 2022 Shah N et al. *JAMA Network Open.*

eFigure: Physicians included in the study

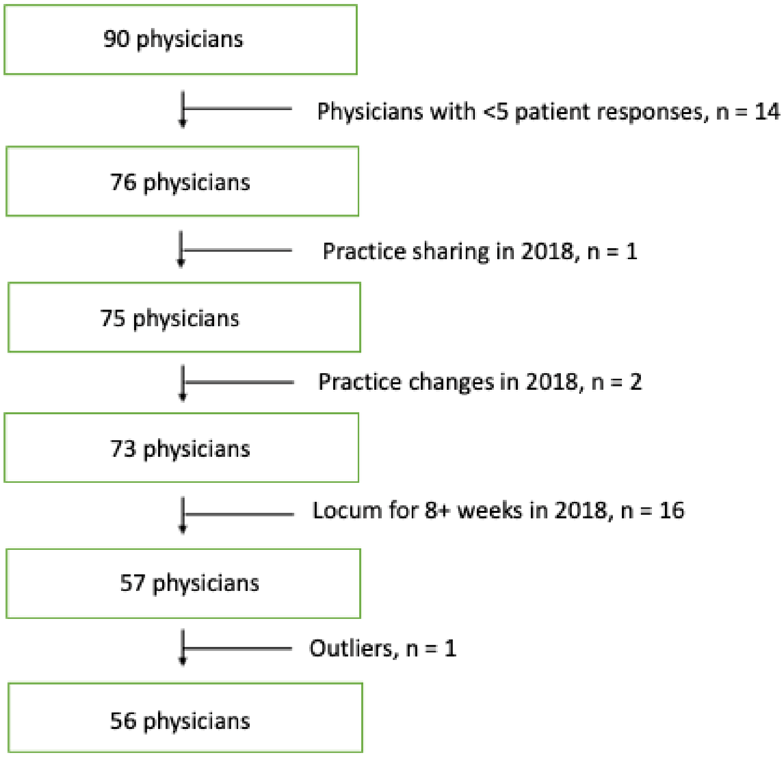

© 2022 Shah N et al. *JAMA Network Open.*
