# Supplementary material for: Association Between Clinic-Reported Third Next Available Appointment and Patient-Reported Access to Primary Care
Source: JAMA Netw Open. 2022 Dec 13;5(12):e2246397. doi: 10.1001/jamanetworkopen.2022.46397 (PMC9856348; doi:10.1001/jamanetworkopen.2022.46397)
Supplement: Supplement 2. — Data Sharing Statement [file jamanetwopen-e2246397-s002.pdf]

Data Sharing Statement

Shah. Association Between Clinic-Reported Third Next Available Appointment and Patient-Reported Access to Primary Care. *JAMA Netw Open*. Published December 13, 2022. doi:10.1001/jamanetworkopen.2022.46397

Data
Data available: No
